# Electrochemical Interrogation of G3-Poly(propylene thiophenoimine) Dendritic Star Polymer in Phenanthrene Sensing

**DOI:** 10.3390/s150922343

**Published:** 2015-09-03

**Authors:** Hlamulo R. Makelane, Oluwakemi Tovide, Christopher E. Sunday, Tesfaye Waryo, Emmanuel I. Iwuoha

**Affiliations:** Sensor Lab, Department of Chemistry, University of the Western Cape, Bellville 7535, South Africa; E-Mails: 3178739@myuwc.ac.za (H.R.M.); ootovide@gmail.com (O.T.); csunday@uwc.ac.za (C.E.S); twaryo@uwc.ac.za (T.W.)

**Keywords:** alternating current voltammetry, cyclic voltammetry, square wave voltammetry, phenanthrene, star-copolymer sensor

## Abstract

A novel dendritic star-copolymer, generation 3 poly(propylene thiophenoimine) (G3PPT)-co-poly(3-hexylthiophene) (P3HT) star co-polymer on gold electrode (*i.e.*, Au|G3PPT-co-P3HT) was used as a sensor system for the determination of phenanthrene (PHE). The G3PPT-co-P3HT star co-polymer was synthesized via *in situ* electrochemical co-polymerization of generation 3 poly (propylene thiophenoimine) and poly (3-hexylthiophene) on gold electrode. 1HNMR spectroscopy was used to determine the regioregularity of the polymer composites, whereas Fourier transform infrared spectroscopy and scanning electron microscopy were used to study their structural and morphological properties. Au|G3PPT-co-P3HT in the absence of PHE, exhibited reversible electrochemistry attributable to the oligo (thiophene) ‘pendants’ of the dendrimer. PHE produced an increase in the voltammetric signals (anodic currents) due to its oxidation on the dendritic material to produce catalytic current, thereby suggesting the suitability of the Au|G3PPT-co-P3HT electrode as a PHE sensor. The electrocatalysis of PHE was made possible by the rigid and planar oligo-P3HT species (formed upon the oxidation of the oligo (thiophene) pendants of the star-copolymer), which allowed the efficient capture (binding) and detection (electrocatalytic oxidation) of PHE molecules.

## 1. Introduction

Phenanthrene (PHE) is listed as one of the polyaromatic hydrocarbon (PAHs) priority chemicals on the Environmental Protection Agency’s (EPA) list. [1]. PAHs are well known as a group of chemicals formed from the incomplete combustion of organic matter such as coal, oil, and wood and fuel, such as diesel and petrol [2,3], and they can find their way into environment sources such as drinking water [4], seawater [5], groundwater [6], and wastewater [7], and also in dietary intake [8]. One of the major driving forces for the development of PHE sensing methods is the growing demand by government and industrial stakeholders to manage the pollution of priority organic pollutants in order to minimize their toxicity [9]. This can be achieved only if adequate technologies are available to determine the occurrence of the PAHs in various sample media. PAHs are known for carcinogenic and mutagenic properties, which pose a high risk for humans coming into contact with polluted sources containing them [10]. Many analytical techniques that are capable of rapid detection of PAHs in environmental samples are widely available and have been well documented. The most commonly used among these techniques include high performance liquid chromatography (HPLC) with ultra violet-diode array detection (UV-DAD) and fluorescence detection (FLD) [11], gas chromatography-mass spectrometry (GC-MS) [12], and high-liquid chromatography coupled with a ultra-violet detector (HPLC-UV) [7]. All these techniques are sensitive and capable of detecting PAHs, however, the limit of detection (LOD) at which the PAHs deleterious to health fall, are not within the detection limits of spectroscopic and chromatographic methods of analysis. Furthermore, many of these analytical techniques require complicated pre-concentrations or multi-solvent extraction steps when used with natural complex samples [13]. Therefore, these methods are not ideal for real-time and on-site monitoring. Electrochemical detection methods, such as anodic stripping voltammetry, have gained substantial popularity in recent years; many of these sensors that have demonstrated high sensitivity, selectivity, and cost-effectiveness, are capable of speciation analysis for PAHs [14,15].

More recently, a new class of PAH sensors, which is based on the amperometric immunosensor using screen-printed electrodes [16] or the dendritic polythiophene electro-catalytic sensor [17] as the sensing motif, has emerged and this sensing strategy has since been reported. Due to the extent of pollution of PAHs in the environment, the need for their continuous monitoring is paramount and this requires the use of simple, low cost, robust and sensitive methods of degradation. The technique that best fits these requirements is the electrochemical sensor. A good way to enhance the electrochemical degradation of a substrate would be to modify the surface of the working electrode used. Because of the conducting properties, dendrimer serves as a good electrochemical membrane for electrode modification [18]. Dendrimer is typically symmetric around the core and often adopts a spherical three-dimensional morphology, which gives it a conductive characteristic that is better than the linear polymer. Conducting chains within the dendrimer and the stereochemistry of the molecule allows sufficient overlap of π-orbitals. This causes the dendritic form of the polymer to have a much higher electrical conductivity than its linear counterpart and the structure also improves sensitivity and selectivity [19].

To our best knowledge, no literature has reported on the levels of the PAHs in wastewater using an electrochemical dendritic sensor with conducting polymer for real time detection of PHE. Dendrimer is considered the most efficient solution to the environmental challenges and is easily synthesized for specific applications [20]; however, it has not been used in the design of this class of PHE sensors. In this study, we used a generation 3 poly(propylene imine) (G3PPI) and conduction polymer poly(3-hexylthiophene) (P3HT) for the fabrication of the star-copolymer (Au|G3PPT-co-P3HT) as the sensing probe. G3PPI has been used as a substrate in the preparation of metal nanocatalysts because of its uniform structure and the ability to protect nanocatalysts’ surfaces from the steric effect, they are also considered to be perfect templates for the preparation of nanocatalysts [21,22]. P3HT on substrates offers a unique strategy for the fabrication of stable, well-defined nanoscale structures that are of potential use as active components in organic field-effect transistors (OFETs) [23]. In particular, the high field-effect mobility and solution-processability of regioregular poly(3-hexylthiophene) (P3HT) strands have stimulated much interest in their utilization as active electronic elements in various thin film devices and sensors [24]. The fabricated star-copolymer was interrogated in both the absence and presence of PHE using cyclic voltammetry (CV), alternating current voltammetry (ACV) and square wave voltammetry (SWV). For the first time, all three techniques were used to systematically characterize a sensor; with the aim of identifying one parameter in each technique that governs the sensor performance in order to obtain the lowest limit of detection (LOD) for PHE. The results presented here not only show that the star-copolymer can be designed and fabricated for the dynamic-based electrochemical PHE sensor, they also highlight sensor specificity, selectivity and sensitivity in detecting PHE.

## 2. Experimental Section

### 2.1. Reagent and Materials

The reagents and materials used in this study included: Generation 3 (G3) poly(propylene imine) (PPI), 3-hexylthiophene (3HT), dichloromethane (DCM), methanol, tetrabutyl ammonium perchlorate [Bu_4_NClO_4_], phenanthrene (PHE), and acetonitrile (CH_3_CN), which were obtained from Sigma-Aldrich (Pty) Ltd., (Sigma-Aldrich, Johannesburg, South Africa) and all chemicals were of analytical grade. NaCl, CuSO_4_, Fe_2_(SO_4_)_3_, MnSO_4_∙H_2_O, NaNO_3_, phenol (PHEN) and petroleum ether (PET) used were obtained from Sigma-Aldrich. Deionized water (18.2 MΩ) purified by a milli-QTM system (Millipore) was used for aqueous solution preparations. Analytical grade argon (African Oxygen Limited (Afrox), Cape Town, South Africa) was used to degas the system. Platinum (Pt) wires as counter electrodes were obtained from Sigma-Aldrich. Micro alumina powder and polishing pads that were used for the polishing of the working electrode were obtained from Buehler (Lake Bluff, IL, USA).

### 2.2. Instruments

All electrochemical experiments were carried out using a BAS 100B electrochemical analyzer from Bioanalytical Systems Inc. (West Lafayette, IN, USA) with a conventional three-electrode system consisting of a gold electrode (Au) as the working electrode, Pt wire as the auxiliary electrode and Ag/AgCl (3 M NaCl) as the reference electrode. The experiments were carried out at controlled room temperature. Fourier transform infrared (FTIR) measurements were done with a Perkin Elmer Spectrum 100-FTIR Spectrometer (Perkin Elmer, Waltham, MA, USA). Scanning electron microscope (SEM) from LEO Gemini 1525 FEGSEM (Carl Ziess, Germany) was used to examine the morphology of the star-copolymer which was coated on the screen printed gold electrode. ^1^HNMR performed with a 200 MHz Varian GerminiXR200 spectrometer (Varian, Inc., Palo Alto, CA, USA) was used to examine G3PPT in CDCl_3_ solvent.

### 2.3. Synthesis of the G3 Poly(propylene thiophenoimine) Dendrimer (G3PPT)

Generation 3 poly(propylene thiophenoimine) (G3PPT) was synthesized by condensation reaction of generation 3 poly(propylene imine) (G3PPI) (0.84 g, 0.51 mmol) with 2-thiophenecarboxaldehyde (2TCA) (0.90 g, 8.02 mmol). This reaction mixture was stirred magnetically in 50 mL dry methanol under nitrogen gas for two days in a 100 mL three-necked round-bottom flask. The removal of the methanol from the reaction mixture was done with a rotator evaporator and residual oil was dissolved in 50 mL dichloromethane (DCM). The organic phase was then washed with 50 mL of water six times to remove unreacted monomer. The solvent was evaporated under reduced pressure to obtain the desired product as yellow oil. The yield of the G3PPT was 0.85 g, 93%.

### 2.4. Electrochemical Preparation of Generation 3 Poly(propylene thiophenoimine)-co-Poly(3-hexlythiophene) Dendritic Star-Copolymer

Prior to modification, the Au electrode was sequentially polished with 1.0, 0.3, and 0.05 μm alumina powder and was rinsed thoroughly with distilled water in-between, followed by sonication in ethanol and water, respectively. Then, 6 µm of 10 mg/mL of G3PPT, already synthesized chemically, were drop coated onto the surface of a polished Au electrode and left to dry at room temperature for 12 h and, hereafter, Au|G3PPT was attained. The modified electrode was immersed into a solution of 0.1 mol/L Bu_4_NClO_4_ in acetonitrile containing 0.1 mol/L 3-hexylthiophene. The CV was run at potential range of −200 to 1800 mV at the scan rate of 50 mV/s for 20 cycles. The P3HT was electrodeposited to the electrode during the polymerization process to form Au|G3PPT-co-P3HT and the electrode was then removed and rinsed with distilled water to remove any traces of monomer. The obtained Au|G3PPT-co-P3HT was kept ready for characterizations.

### 2.5. Characterization of Dendritic Star-Copolymer Au|G3PPT-co-P3HT

A three-electrode system was used for electrochemical characterization of the polymerized star-copolymer Au|G3PPT-co-P3HT. A modified Au electrode with G3PPT-co-P3HT was used as the working electrode, platinum wire as the auxiliary electrode, and Ag/AgCl (3 mol/L NaCl) as the reference electrode. The characterization solution used contained 5 mL of 0.1 mol/L Bu_4_NClO_4_ in acetonitrile added into an electrochemical cell and degassed for about 5 min using argon gas. CV, SWV and ACV used for Au|G3PPT-co-P3HT characterization in the above solution.

### 2.6. Electrochemical Degradation of Phenanthrene

All three electrochemical techniques were used in sensor interrogation. The calibration curves were obtained by sequential addition of increasing concentration of PHE in the solution of acetonitrile and water in the ratio 80:20 (acetonitrile:water). This mixture of acetonitrile and water was used as the solvent in all sensor applications for PHE degradation. The sensor was allowed to equilibrate for 15 min after each addition of PHE prior to collecting the voltammograms and unless stated, magnetic stir at 1000 rpm was applied at all times.

### 2.7. Sensor Specificity and Selectivity

Several substances suspected to interfere with the detection of PHE were investigated by the developed electrochemical sensor. These substances included inorganic substances, such as Na^+^, Cu^2+^, Fe^2+^, Mn^2+^, Cl^−^, SO_4_^2−^, NO_3_^−^ and NO_2_^−^ [25] and organics species, such as chlorinated benzenes, nitrogen compounds, phenol and petroleum ether [26]. The standard solution of 1 µmol/L organic compounds, Na^+^, Mn^2+^, Cu^2+^, Fe^2+^, CI^−^, SO_4_^2−^, and NO_3_^−^, and the organic compounds phenol and petroleum ether, were prepared. From these stock solutions, aliquots (15 μL) were drawn sequentially and added to the cell for specificity and selectivity investigation. All compounds were initially analyzed separately in the absence of PHE to monitor its effect on the sensor and in the presence of 1 µmol/L PHE with ratio 1:1. The inorganic species used were combined into anions (CI^−^, SO_4_^2−^, NO_3_^−^) and cations (Na^+^, Mn^2+^, Cu^2+^, Fe^2+^) and the solutions analyzed in the absence and in the presence of PHE. Finally, all the species were combined and analyzed without and with PHE. The sensor was allowed to equilibrate in the presence of the specific compound until no change in the current was observed and voltammograms were then collected using all three techniques. PHE was added to the solution to confirm that the sensors were still functional after interrogation with all of the above compounds. For real sampling, tap water was used in this study for sensor determination of PHE. Tap water samples were collected at the laboratory located in the chemistry department of the University of the Western Cape. Water samples with spiked PHE were analyzed using the three electrochemical techniques.

## 3. Results and Discussion

### 3.1. Dendritic Star-Copolymer (Au|G3PPT-co-P3HT) Sensor Modification

**Scheme 1:**
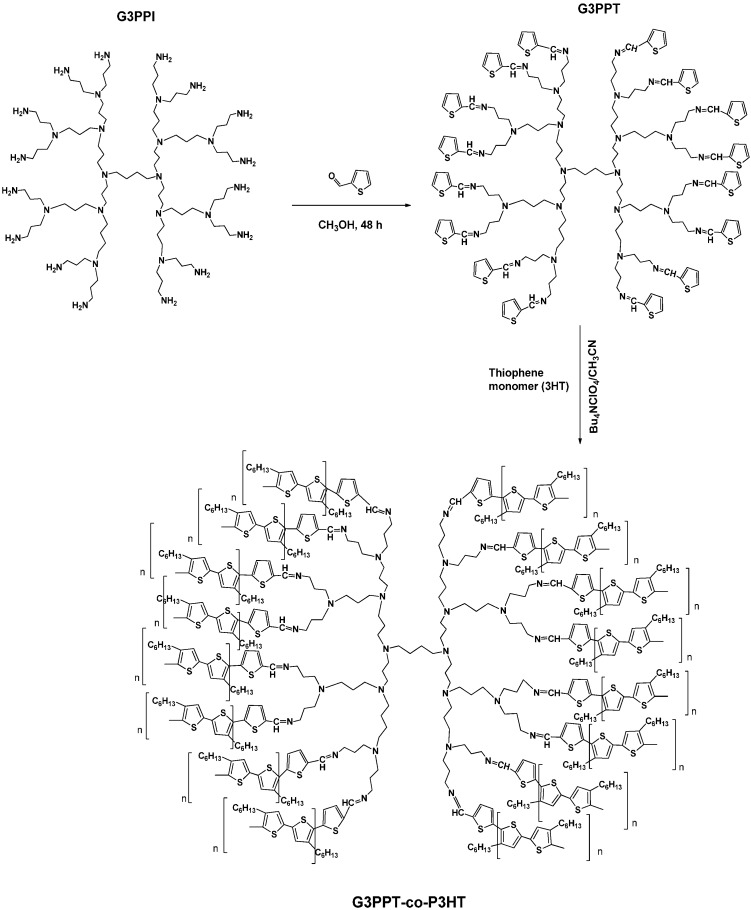
Synthesis of generation three poly(propylene thiophenoimine) dendrimer.

The first focus of the research presented here was the peripheral modification of this G3PPI dendrimer. The synthesis of the G3PPT dendrimer was prepared by the condensation reaction of G3PPI with 2TCA following a procedure reported in the literature [27]. Generation 3 PPI dendrimer was initially chosen for this study due its reactive periphery and cavities (define hosting location). The G3PPI dendrimer terminated with primary amine end-group produces a highly reactive periphery for easy conversion. The reactive end-group moiety also allowed the thiophene functional group to be attached to the dendrimer through a simple coupling reaction (Scheme 1).

**Figure 1:**
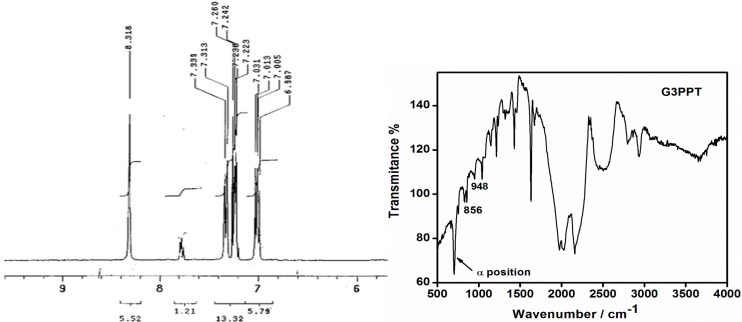
^1^HNMR and FTIR spectrum of generation 3 poly(propylene thiophenoimine) (G3PPT).

In the ^1^HNMR spectra of G3PPT, the characteristic presence of a singlet *δ* *ca*. 8.30 ppm, which corresponds to CH=N, and the appearance of peaks at *δ* *ca*. 6.31–7.34 ppm which correspond to the thiophene hydrogen group signals in the aromatic region was observed, as shown in Figure 1 (full spectra Figure S1), distinguish G3PPT. This leads to the conclusion that amines form a covalent bond with the alkoxide precursor via carbon–nitrogen bonds because of the resonance interaction of the nitrogen unshared pair with the double bond bands arising at *v*max *ca*. 856 and 948 cm^−1^ [28]. The peak at *v*max *ca*. 706 cm^−^^1^ is due to α position of thiophene moiety (Figure 1). The spectroscopic data (^1^HNMR and FTIR) for the G3PPT are consistent with the predicted structure.

**Figure 2:**
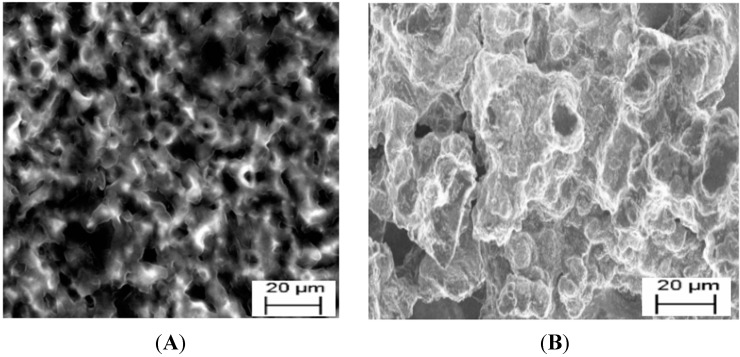
Scanning electron microscopic images of (**A**) G3PPT-co-P3HT; (**B**) P3HT.

The star-copolymer sensor (Au|G3PPT-co-P3HT) was constructed with controllable periphery that could be manipulated by some stimuli in order to regulate the containment/egress of the guest molecule (PHE). The stimuli-responsive molecule that will allow this type of control was a P3HT moiety. P3HT has the ability to oligomerize through oxidation and reduction techniques having the electrical conductivity application property [29]. This redox-active, oligomer P3HT species possesses two distinct conformations, which are seen to be the controlling entity for G3PPT dendrimer system by studying the scanning electron microscopy (SEM) images in Figure 2A. A rigid, planar oligo(P3HT) species was formed upon oxidation of the oligothiophene-terminated dendrimer to allow the guest molecules to be captured, while the reduced species has freedom of rotation that is seen to enhance egress of the guest molecules.

A different morphology was also observed from the dendritic star-copolymer, which indicated the enlargement of the globular structures of the dendrimer moiety growing through the available holes of P3HT film. P3HT moiety morphology in Figure 2B reveals the growth of P3HT in a sponge-like structure and branched polymer with the surface having shallow pores. From this observation, it can be concluded that dendritic star-copolymer was symmetrical and the patterns of the pores on dendritic star-copolymer were converted by the presence of P3HT in order to regulate the PHE in the solution as the contaminant/guest for detection.

### 3.2. CV and ACV Responses of Sensor

**Figure 3:**
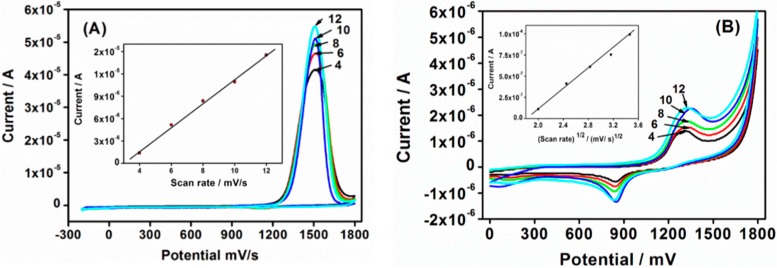
CV voltammograms (**A**) Scan rate dependence of Au|G3PPT-co-P3HT sensor recorded in the absence of PHE in 0.1 mol/L Bu_4_NClO_4_/CH_3_CN; (**B**) The CV scan rate dependence in the presence 3.0 × 10^−3^ µmol/L PHE in 0.1 mol/L Bu_4_NClO_4_/CH_3_CN/H_2_O, (numbers on the diagrams are scan rate mV/s.)

The CV technique is one of the most commonly used for sensor interrogations. It was first used in this study to characterize the Au|G3PPT-co-P3HT star-copolymer sensor. The comparison of star-copolymer with the bare electrode showed the difference in the peak enhancement in the absence and presence of PHE (Figure S2). A higher anodic peak current was obtained with dendritic star-copolymer compared to that of the bare gold electrode. The significant response occurred for Au|G3PPT-co-P3HT modified electrode was attributed to the combination of the unique properties exhibited by the functionalized dendrimer and the conducting polymer. A similar observation was obtained with ACV and SWV. Figure 3A shows CV voltammograms scan rate dependence of Au|G3PPT-co-P3HT sensor recorded in the absence of PHE. The anodic peak current increased as the scan rate increased, indicating that the peak current depends on the scan rate. At the scan rate of 4 mV/s, lower peak current 4.01 × 10^−5^ A was observed at the potential ~+1500 mV, whereas, a higher peak current of 5.78 × 10^−5^ A at ~+1500 mV was observed at a higher scan rate of 12 mV/s. The results observed were due to increased surface area of the Au|G3PPT-co-P3HT modified electrode and the enhanced conductive characteristics of the dendritic star-copolymer [30,31]. The peak current also occurred at the same voltage attributing to the characteristic of star-copolymer electrode reactions, which have rapid electron transfer kinetics. This indicates that the star-copolymer has a reversible behavior. The peak current linearly increases with an increase in potential scan rate as shown in Figure 3A (inserted plot). It indicates that the Au|G3PPT-co-P3HT platform is diffusion controlled [32]. An estimation of the surface coverage of the electrode, *Γ*, was determined from the slope line of peak current *versus* scan rate (inserted in Figure 3A). The value was estimated to be about 4.2 × 10^−8^ mol/cm^2^, according to Equation (1) [33]. Based on this method, the peak current is related to the surface concentration of the electroactive species. This also confirms that the dendritic star-copolymer is confined to the surface of the electrode and it is electroactive with conductivity [34].

(1) $$i_{p}=n^{2}F^{2}A\Gamma \nu /4\text{RT}$$

where *n* is the number of electrons involved in the reaction, *A* is the surface area (2.01 × 10^−2^ cm^2^), *F* is the Faradays constant (96486 C/mol), Γ is the surface coverage of the electrode, and R is the ideal gas constant.

The effect of the scan rate response of the Au|G3PPT-co-P3HT sensor in the presence of 3.0 × 10^−3^ µmol/L PHE was investigated. Figure 3B shows the cyclic voltammograms at different scan rates, and the CV voltammograms of the oxidation peak current increasing with the increase in scan rate, ranging from 4 to 12 mV/s; this was similar to the one observed in the absence of PHE. The peak current increased linearly with square root of scan rate in accordance to the linear regression equation of *I_p,a_* (A) = 5.79 × 10^−7^ *v*^1/2^ + 1.03 × 10^−6^ (correlation coefficient, *r*^2^ = 0.996) (inserted in Figure 3B). This indicated that the electrochemical process was limited by the rate of diffusion of PHE from the solution to the star-copolymer sensor surface, and the peak potential shifted slightly to more positive, thereby confirming that the peak currents were diffusion controlled [35,36].

**Figure 4:**
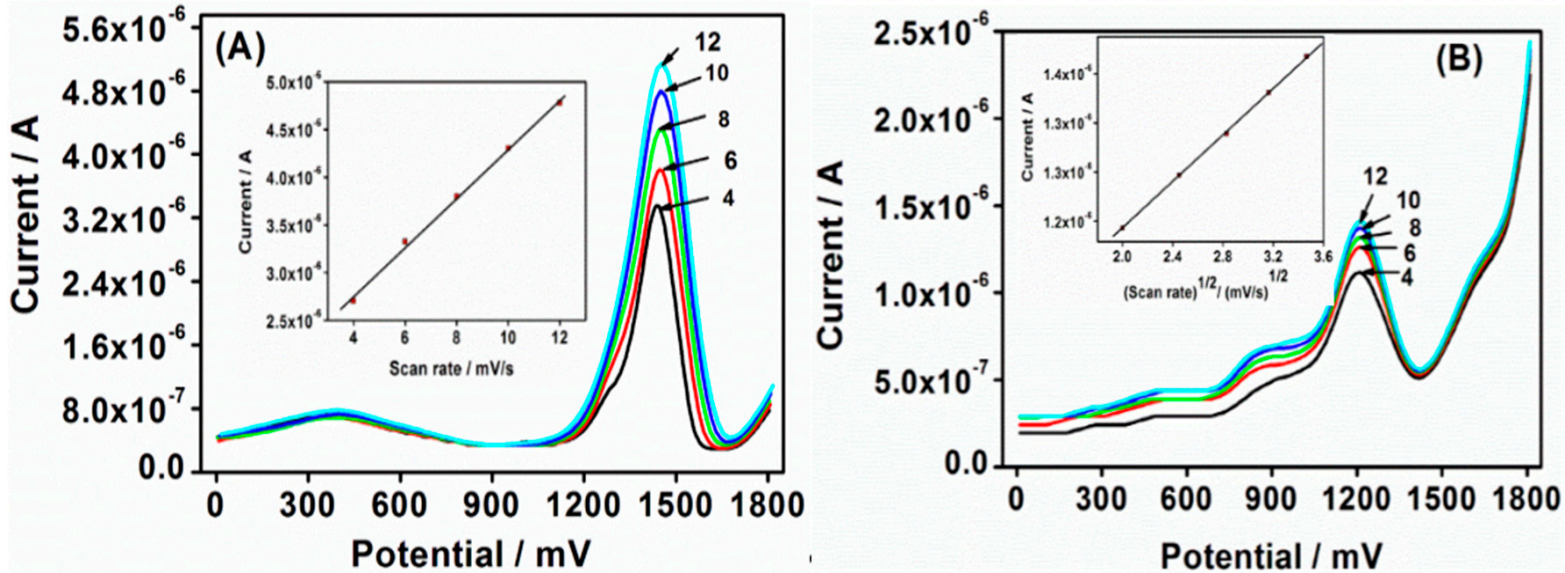
(**A**) AC voltammograms scan rate dependence of Au|G3PPT-co-P3HT sensor recorded in the absence of PHE in 0.1 mol/L Bu_4_NClO_4_/CH_3_CN; (**B**) The AC voltammograms scan rate dependence in the presence 3.0 × 10^−3^ µmol/L PHE in 0.1 mol/L Bu_4_NClO_4_/CH_3_CN/H_2_O (numbers on the diagrams are scan rate mV/s.)

Although CV is most commonly used in sensor characterization, ACV was employed for star-copolymer characterizations because ACV is a potential sweep method, which is very similar to CV [37]. The effects of the star-copolymer in the absence of PHE under similar conditions were investigated as before. In the recorded voltammograms in Figure 4A, the AC peak current also shows dependence on the scan rate, and an increase in the scan rate caused an increase in the peak current. The linearity of the peak current with scan rate was an important diagnostic criterion for surface attachment of the star-copolymer. The peak current for dendritic star-copolymer increased linearly with the increase in scan rate over 4–12 mV/s with linear regression of *I_p,a_* (A) = 2.27 × 10^−7^ *v* + 2.46 × 10^−6^ (*r*^2^ = 0.998). This indicates that the diffusion process has an effect at the star-copolymer film. Moreover, the linear relationship between scan rate and peak current also confirmed that the dendritic star-copolymer was confined to the surface of the electrode and was electroactive and conductive [34]. When the scan rate was changed, no shift in peak potential was observed. This indicates that the rate of the electrode process increased in the supporting electrolyte solution and also confirms some reversibility of the redox process of the dendritic star-copolymer on the electrode with an effective interfacial accumulation [38].

In the presence of PHE, the effect of the scan rate on the star-copolymer Au|G3PPT-co-P3HT sensor shows the AC voltammogram of the oxidation peak current increasing with the increase in scan rate ranging from 4 to 12 mV/s (Figure 4B). The anodic peak current increased linearly with root scan rate in accordance with the linear regression equation of *I_p,a_* (A) = 1.42 × 10^−6^ *v*^1/2^ + 1.83 × 10^−7^ (correlation coefficient, *r*^2^ = 0.999). This indicated that the electrochemical process was limited by the rate of diffusion of PHE from the solution to the star-copolymer sensor surface.

The CV and ACV voltammograms of the star-copolymer sensor in the absence and in the presence of PHE are shown in Figure S3 in supporting data. In the presence of 3.0 × 10^−3^ µmol/L PHE, the star-copolymer exhibited peak current enhancement of approximately 90% compared to the peak current in the absence of PHE. It is noteworthy that a higher anodic peak current of star-copolymer in the presence of PHE was attributed to the presence of G3PPT and P3HT in the fabricated star-copolymer. This changed the electrode surface as well as the transfer rate of electrons, which resulted in the oxidation of PHE on the star-copolymer, showing a better electrocatalytic performance with a higher catalytic performance. It also indicates that the variable multi-functionality and intrinsic architecture of G3PPT dendrimer with internal cages strongly stabilized the dendritic star-copolymer [39]. Additionally, the high density of catalytically active species at the periphery of the dendritic support is expected to lead to synergistic effects and selectivity of the PHE [40].

### 3.3. SWV Responses of Sensor

**Figure 5:**
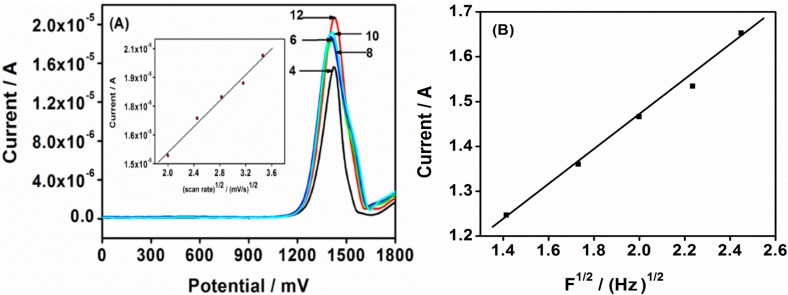
AC voltammograms (**A**) Scan rate dependence of Au|G3PPT-co-P3HT sensor recorded in the absence of PHE in 0.1 mol/L Bu_4_NClO_4_/CH_3_CN (experimental conditions were: the SW frequencies of the scan rates (mV/s) were 2 (4), 3 (6), 4 (8), 5 (10), and 6 Hz (12); and the other experimental conditions were: SW amplitude *E*_sw_ = 25 mV and scan increment *E*_s_ = 2 mV); (**B**) The dependence of peak current on the square root of the frequency for AuǀG3PPT-co-P3HT.

Under the aforementioned experimental conditions in this study, the Au|G3PPT-co-P3HT star-copolymer modified electrode was observed to be a good PHE electrochemical sensor when using both CV and ACV electrochemical interrogation techniques. This observation is supported by the results discussed under the sections on CV and ACV. The dependence of net peak current and peak potential on the star-copolymer were investigated in the absence and in the presence of PHE. The parameters, such as pulse amplitude, frequency and step potential increment, were carefully applied because these parameters interrelate and have a combined effect on the peak current [41]. Thus, optimization was realized in such a manner that each parameter was changed while the others were kept constant, as illustrated in Figure 5A. The different scan rates were obtained by Δ*E*_base_ × S.W. frequency (step increment × frequency). These two parameters, Δ*E*_base_ and S.W. frequency, define the scan rate with fixed pulse amplitude, and the peak current responded proportionally to the scan rate. This observation confirms that the electrochemical reaction at the Au|G3PPT-co-P3HT star-copolymer modified electrode is diffusion controlled [42]. A linear dependence of anodic current on the scan rate was observed when a plot of peak current *versus* the square root of scan rate with a correlation coefficient of 0.993 was observed (inserted in Figure 5A). The observed result indicates that the star-copolymer is a surface-bound electroactive species undergoing fast electron transfer reaction at the electrode. The diffusion coefficient of the electron (*D*), which is the rate of electron transport within the star-copolymer, was obtained to be 6.45 × 10^−5^ cm^2^/s using the Randles-Sevcik equation (Equation (2)) [27]:

(2) $$I_{p}=269n^{3/2}AD^{1/2}v^{1/2}C$$

where *I_p_* is peak current, *n* is number of electrons, *A* is the area, *D* is diffusion coefficient, *v* is the scan rate, and *C* is bulk concentration of the solution.

The reversibility of the dendritic star-copolymer electrode was analyzed. The reversibility of redox peaks varied experimentally by adjusting the frequency of the signal with the SW properties. The response of a reversible electrode occurring in a limiting diffusion space and mainly controlled by the film thickness was obtained. Figure 5B shows the peak current ratio *versus* the corresponding square root of frequency. For the dendritic star-copolymer, the peak current depends linearly on the square root of the frequency) confirming the film thickness and the reversibility of Au|G3PPT-co-P3HT, which was also confirmed by CV. The relationship between the peak current and the SW frequency depends on the thickness of the film. The dependence of peak current on the applied frequency is in agreement with the theoretical electrode study by Lovrić *et al.* [43]. However, there are only a limited number of reactions within the available range of frequencies that can undergo such transformation. It should be noted that increase in frequency will affect the thickness parameter simultaneously. The increase in peak current indicates that the diffusion length is equal to the film thickness, *i.e.* when *L* = (*D/f*)^1/2^ [44]. From Equation (3), thickness layer was determined and obtained to be >10 µm with a diffusion coefficient of 6.45 × 10^−5^ cm^2^/s at the frequency range of 2 to 6 Hz. Equation (4), was applied to determine thickness parameter, which was obtained to be *Ʌ* ≤ 0.3 [44]. This indicates that the star-copolymer is a characteristic of reversible electroactive thin film, and for thin films, the peak current ratio of the forward and backward components is sensitive to the frequency. This is in agreement with the theoretical electrode study by Mirceski *et al.*, which reported that, for a thin film, *Ʌ* ≤ 0.949 [44].

(3) $$L={\left(\;D/f\right)}^{1/2}$$

(4) Λ=LfD

where *L* is thickness of the layer, *D* is the diffusion coefficient that was used to determine *L*, *f* is the SW frequency, and *Ʌ* is thickness parameter.

### 3.4. Application of the Phenanthrene Sensor

**Figure 6:**
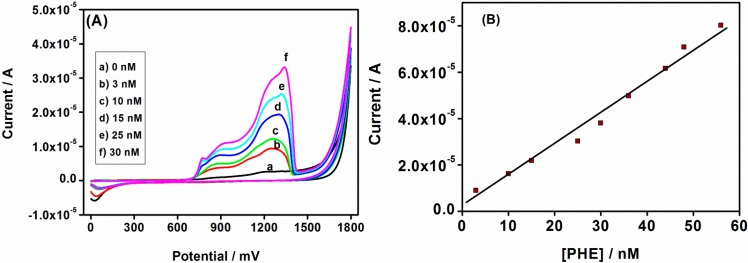
(**A**) CV voltammograms of the Au|G3PPT-co-P3HT in 0.1 mol/L Bu_4_NClO_4_/CH_3_CN/H_2_O with different PHE concentrations; (**B**) The calibration plot of Au|G3PPT-co-P3HT sensor for PHE.

CV, ACV and SWV techniques were used to study the electrochemical responses of the Au|G3PPT-co-P3HT phenanthrene sensor in acetonitrile/water solution containing 0.1 mol/L Bu_4_NClO_4_ as the supporting electrolyte. The effects of changes in concentrations of PHE were studied with CV, and the observed voltammograms show that the peak current increased as PHE concentration increased and the peak current of PHE appeared at slightly more positive potentials (Figure 6A) than the peak current observed without PHE, indicating the oxidation of PHE. The anodic peak at ~+1285 mV with no corresponding reduction peak, is ascribed to the oxidation of PHE on the star-copolymer electrode. This indicated the oxidation of PHE and also confirmed the catalytic behavior of the star-copolymer in detecting PHE. Thus, in every addition of PHE concentration, the anodic peak increased until it reached stabilization and this was because of the terminal residues of dendrimer modification on the electrode surface that play a role in the sensor stability. The sensor stability is also determined by the pores in the dendritic star-copolymer becoming saturated by PHE oxidation. The dendrimer structure is known for being porous which plays an important role on the electrode surface by binding strictly to gold metal ions and ensuring that particles of almost the same size are obtained, using the availability of the porous structure [45]. The calibration curve of the PHE sensor is shown in Figure 6B. A good linear relationship between anodic peak current and PHE concentration was obtained over a 6.68–40.79 nmol/L range with coefficient *r*^2^ = 0.990, and the LOD found to be 4.74 nmol/L (calculated from the six times standard deviation of blank measurements).

**Figure 7:**
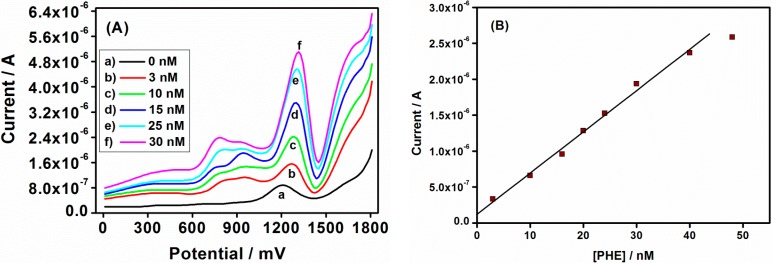
(**A**) AC voltammograms of Au|G3PPT-co-P3HT in 0.1 mol/L Bu_4_NClO_4_/CH_3_CN/H_2_O with different PHE concentrations; (**B**) The calibration plot of Au|G3PPT-co-P3HT sensor for PHE.

For the ACV effect of PHE concentration, a similar observation as the CV was obtained, as shown in Figure 7A. The obtained peak currents appeared at slightly high potentials compared to the peak current without PHE. The observed peaks indicated the unique role played by the conducting polymer (P3HT) regarding the sensitivity and selectivity of the composite systems with respect to the determination of PHE in the solution [46,47]. The calibration curve of anodic current *versus* concentration of PHE, shown in Figure 7B, describes the relation of peak current to the PHE concentration. The peak current was observed to increase with the increase in PHE concentration, and a linear relationship between the anodic peak current and PHE concentration was obtained over a range of 4.78–37.65 nmol/L with correlation coefficient of *r*^2^ = 0.996. The LOD of PHE was found to be 1.42 nmol/L, which was lower than the calculated one from the CV voltammograms.

**Scheme 2:**
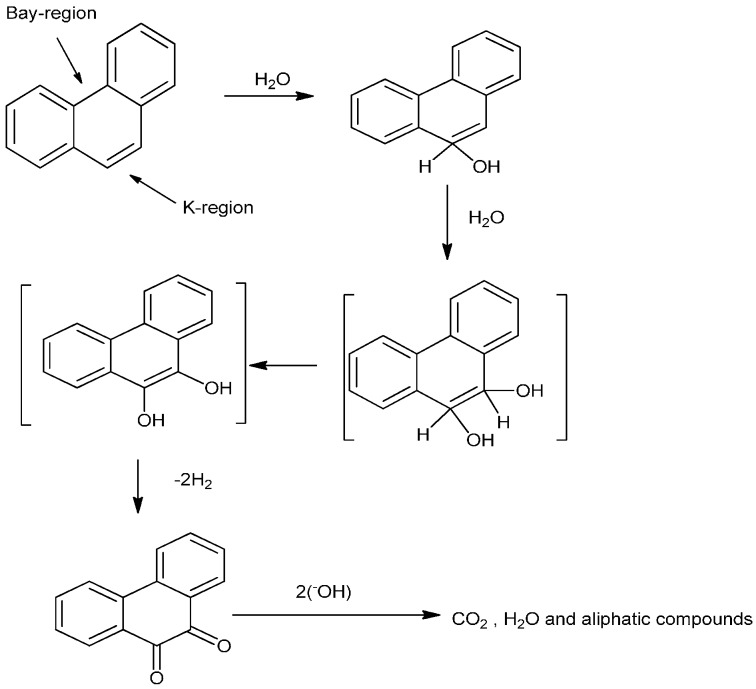
Proposed phenanthrene degradation pathway.

**Figure 8:**
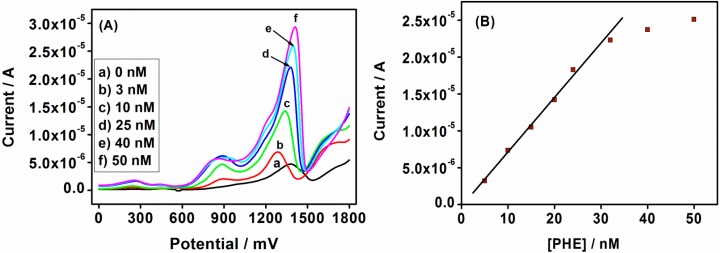
(**A**) SWV voltammograms of the Au|G3PPT-co-P3HT in 0.1 mol/L Bu_4_NClO_4_/CH_3_CN/H_2_O with different PHE concentrations; (**B**) The calibration plot of Au|G3PPT-co-P3HT sensor for PHE (experimental conditions were: the SW frequencies = 5 Hz, scan increment *E*_s_ = 2 mV and SW amplitude of *E*s_w_ = 25 mV).

The electrochemical behavior of star-copolymer with SWV revealed the formation of an anodic peak on the star-copolymer modified electrode and an increase in the anodic peak was obtained upon addition of PHE (Figure 8A). These peaks revealed that the PHE oxidized first in the interior of the dendrimer periphery surface functional groups, which serve as controlling gates for the entry or departure of PHE molecules to or from the dendrimer interior [48]. The shift in peak potential was slightly less positive than with AC and ACV. This observation led to the evaluation of the proposed degradation pathway of phenanthrene (Scheme 2). The PHE structure consisted of two regions, the bay- and K-region, which was attributed to the observed peaks at different potentials for PHE oxidation, in which either the K- or bay-region might be oxidized first in the interior of the dendrimer. Moreover, the shift in peak potential might be attributed to the hydrogen emission during PHE oxidation. The plot of peak current *versus* PHE concentration in Figure 8B shows a good linear relationship between anodic peak current and the PHE concentration over the range of 5.35–38.86 nmol/L with correlation coefficient *r*^2^ = 0.985, giving the LOD of 3.24 nmol/L. The least that can be concluded is that CV, ACV and SWV with dendritic star-copolymer sensor exhibited the possibility of the PHE degradation taking place during oxidation [49].

### 3.5. Sensor Specificity

**Figure 9:**
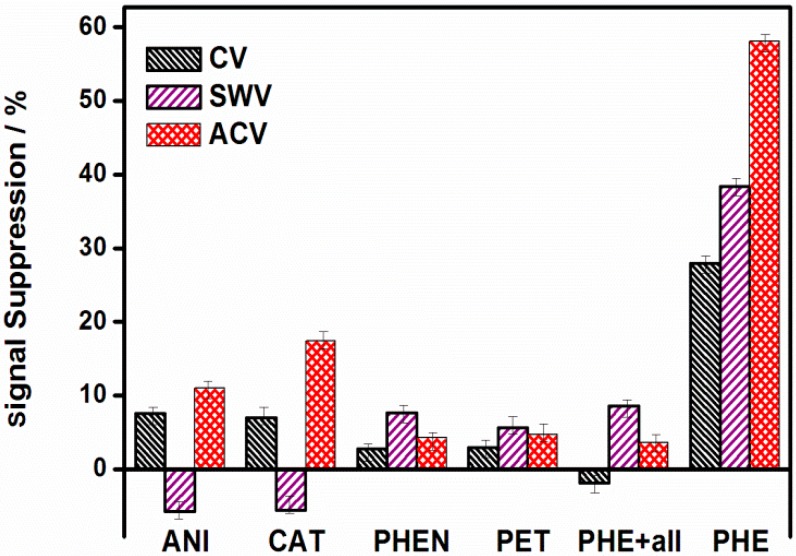
Sensor response to 1 µmol/L ANI (CI^−^, SO_4_^2−^, NO_3_^−^), CAT (Na^+^, Mn^2+^, Cu^2+^, Fe^2+^), PHEN, PET, 1 µmol/L PHE with all inorganic/organic species (in a ratio of 1:1) interrogated using CV, ACV and SWV.

To determine sensor specificity, the star-copolymer sensor was interrogated with different metal ions, including Na^+^, Cu^2+^, Fe^2+^, CI^−^, SO_4_^2−^, and NO_3_^−^ and with organic species including phenol (PHEN) and petroleum ether (PET) in 0.1 mol/L Bu_4_NClO_4_/CH_3_CN/H_2_O, using all three electrochemical techniques. It is worth repeating that each inorganic and organic species was analyzed separately, the inorganic species used were also combined into anions (ANI) and cations (CAT), and all the species combined were also analyzed to monitor their effect in the absence and in the presence of PHE (the concentration used was 1 µmol/L). From the data obtained from the experimental conditions, the signal suppression was determined using Equation (5) [50] to obtain the data shown in Figure 9. %SS was observed to be slightly higher with ACV in the presence of ANI and CAT, when compared with the %SS with CV and SWV. With the addition of all species, the %SS was obtained to be slightly lower in ACV and CV. Sensor specificity appeared to be suppressed less when SWV was used; the extent of signal change in the presence of all species was slightly higher than that obtained when species were analyzed separately. For example, the addition of all species with PHE resulted in ~8.7%SS, ~3.9%SS and ~−1.8%SS, respectively. The specificity results were, in fact, comparable to those shown in ACV and CV. In addition, the adsorption of matrix contaminants could potentially contribute to the reduction in the redox signal. Overall, the star-copolymer sensors performed optimally when interrogated using ACV and SWV as evidenced by the large %SS in the presence of the PHE.

ACV compared to SWV appears to be the inferior “signal-on” interrogation technique due to the lower %SS. The %SS observed with PHE was significantly higher than that observed with other species. Overall, the results shown here suggest that the sensor is highly specific.

(5) $$\text{Signal Suppression }(\%)=\left[\frac{\left(I_{o}-\;I\right)}{I_{o}}\right]\;\times 100\text{\%}$$

where $I_{o}$ represents the dendritic star-copolymer current in the absence of PHE, and I represents the current in the presence of PHE.

### 3.6. Sensor Sensitivity and Selectivity

Sensor selectivity and sensitivity are equally important factors that govern the sensor’s suitability for real world applications [51]. Phenanthrene is found in environmental samples, such as drinking water, wastewater, seawater and ground water [52,53,54,55]. The World Health Organization (WHO) stated that some PAHs are known to be carcinogenic and that the concentrations should therefore not, in general, exceed (0.0002 mg/L) in drinking water [56]. It was reported by May *et al.* [57] that the solubility of PHE in water is 1002 µg/L (5.62 µmol/L). In this study, ACV, CV and SWV were used to evaluate sensor sensitivity in the real sample and in determining the LOD. As shown in Table 1, the sensor responded well to the added PHE concentration in tap water. The observed peak current of PHE in tap water was compared to that of PHE added to the supporting electrolyte. The peak current in tap water solution was observed to be lower than the one observed in the supporting electrolyte. This was attributed to the presence of other anions capable of complexing PHE in the tap water sample, as discussed under sensor specificity. The same trend was also observed in SWV and CV in which the peak current was lower when the sensor was interrogated in tap water samples. From the three techniques investigated; comparisons were made for detection of PHE. From CV the LOD was obtained to be higher than the other techniques. In ACV, the sensor resulted in giving the lowest LOD for PHE concentration (Table 1). The obtained LODs from the star-copolymer PHE sensor using all three techniques were compared with other sensors from the literature. The Au|G3PPT-co-P3HT sensor is more sensitive than the reported PAH sensors; dendritic polythiophene electro-catalytic sensor gave the LOD of 19.0 nmol/L [17] and the amperometric immunosensor using screen-printed electrodes gave the LOD of 4 ng/mL (0.022 µmol/L) [16].

In this study, three techniques were used to evaluate sensor selectivity in standard solution and waster sample. Signal enhancement (%SE) was determined for sensor selectivity using Equation (6) [51] and the lowest %SE of the sensor was obtained with SWV, with ~20%SE, and, with CV, the %SE obtained was ~74%. However, ACV resulted in a %SE of ~84%, which was the highest. The signal enhancement in tap water was also determined and it resulted in ACV giving ~68%SE as shown in Table 1. The lower %SE could be attributed to the presence of other anions capable of complexing PHE in the sample. With all the investigations and interrogation of the sensor from the three different techniques, the best parameters observed favored the ACV technique for the dendritic star-copolymer phenanthrene sensor.

(6) $$\text{Signal enhancement }(\%)=\left[\frac{\left(I-\;I_{o}\right)}{I_{o}}\right]\;\times 100\text{\%}$$

where $I_{o}$ represents the dendritic star-copolymer current in the absence of PHE, and I represents the current in the presence of PHE.

**Table 1:** CV, ACV and SWV parameters of the Au|G3PPT-co-P3HT phenanthrene sensor

Techniques	Solutions	R^2^	LOD (nmol/L)	Linear Range (nmol/L)	%SE
CV	0.1 MBu_4_NClO_4_/CH_3_CN/H_2_O	0.990	4.74	6.68–40.79	74
	Tap water	0.998	12.62	13.35–35.86	11
ACV	0.1 MBu_4_NClO_4_/CH_3_CN/H_2_O	0.997	1.42	4.78–37.65	84
	Tap water	0.983	2.99	5.46–42.57	68
SWV	0.1 M Bu_4_NClO_4_/CH_3_CN/H_2_O	0.985	3.24	5.35–38.76	20
	Tap water	0.988	9.61	10.34–40.56	7

## 4. Conclusions

In this study, a simple and sensitive Au|G3PPT-co-P3HT sensor was developed for the detection of PHE. The results demonstrated that dendritic G3 poly(propylene thiophenoimine) with polythiophene enhanced the stability and the performance of the star-copolymer sensor for PHE determination. This was due to a rigid, planar oligo-P3HT species formed upon the oxidation of the oligothiophene-terminated dendrimer allowing PHE molecules to be captured/detected. The Au|G3PPT-co-P3HT sensor was interrogated electrochemically using CV, ACV and SWV. The three electrochemical techniques indicated that the star-copolymer had excellent conductivity. This is attributed to the increase in the conjugation length of the copolymer after the incorporation of the P3HT onto the dendrimer, thereby facilitating charge transfer through the star-copolymer. Preliminary results from this study indicate that the sensor can be deployed for PAHs analysis in water systems.

## Supplementary Files

Supplementary File 1
