# Supplementary material for: Electrochemical Interrogation of G3-Poly(propylene thiophenoimine) Dendritic Star Polymer in Phenanthrene Sensing
Source: Sensors (Basel). 2015 Sep 3;15(9):22343–63. doi: 10.3390/s150922343 (PMC4610539; doi:10.3390/s150922343)
Supplement: Supplementary File 1 [file sensors-15-22343-s001.pdf]

OPEN ACCESS
sensors
ISSN 1424-8220
www.mdpi.com/journal/sensors

Supplementary Information

Electrochemical Interrogation of G3-Poly(propylene thiophenoimine) Dendritic Star Polymer in Phenanthrene Sensing. *Sensors* 2015, *15*, 22343-22363

Hlamulo R. Makelane, Oluwakemi Tovide, Christopher E. Sunday, Tesfaye Waryo and Emmanuel I. Iwuoha *

Sensor Lab, Department of Chemistry, University of the Western Cape, Bellville 7535, South Africa; E-Mails: 3178739@myuwc.ac.za (H.R.M.); ootovide@gmail.com (O.T.); csunday@uwc.ac.za (C.E.S); twaryo@uwc.ac.za (T.W.)

* Author to whom correspondence should be addressed; E-Mail: eiwuoha@uwc.ac.za; Tel.: +27-219-593-054; Fax: +27-219-593-055.

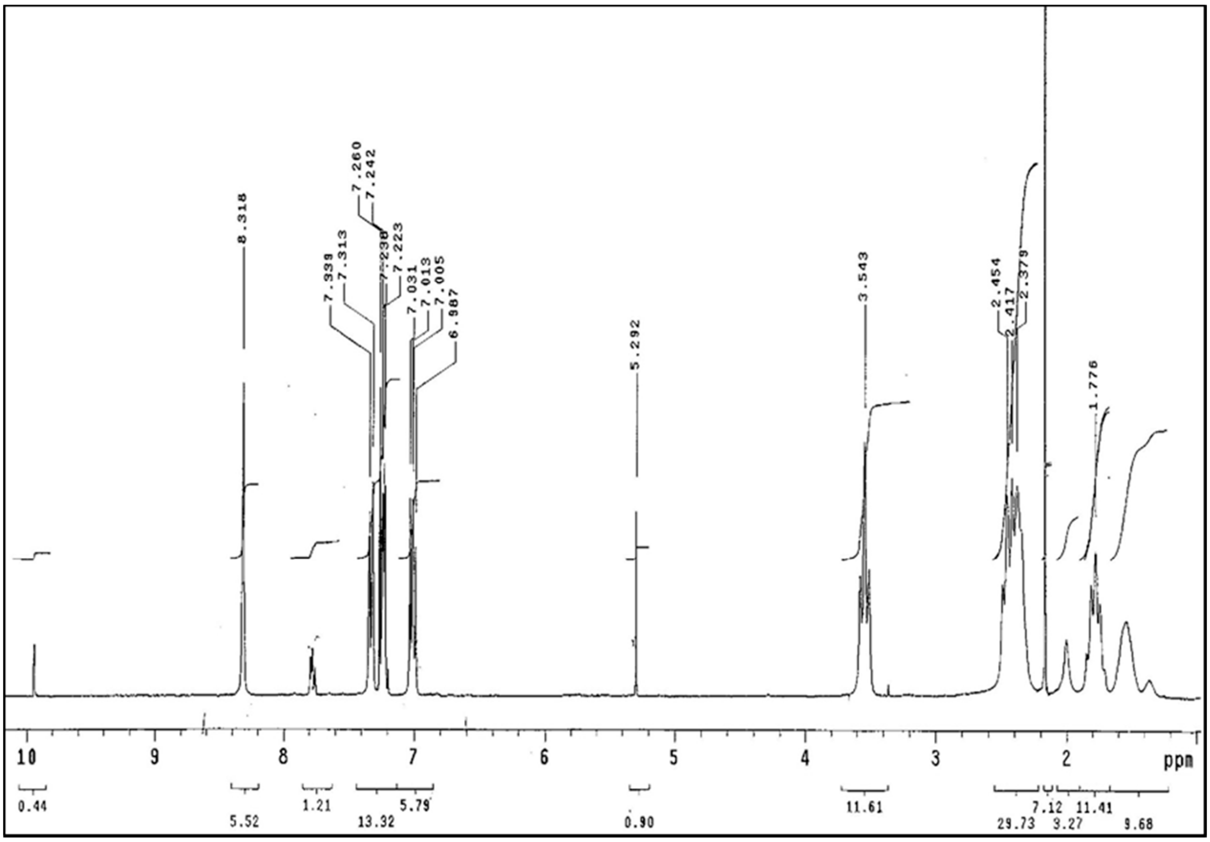

Figure S1. Fourier transforms infrared spectra of G3PPT.

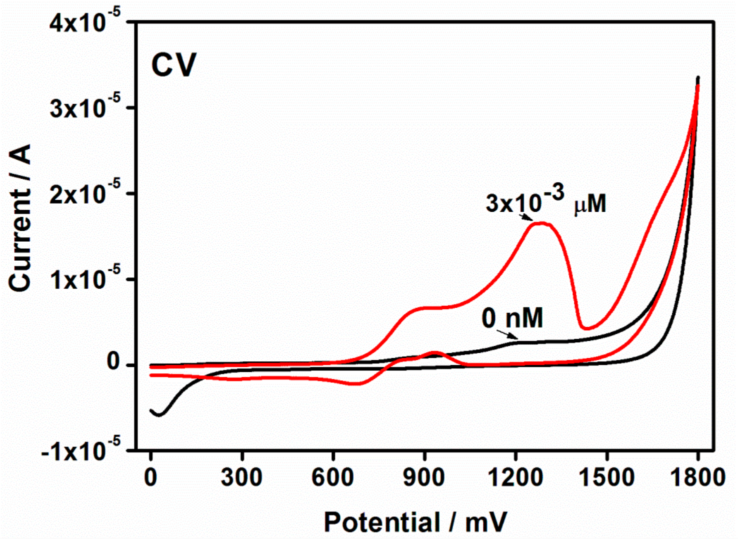

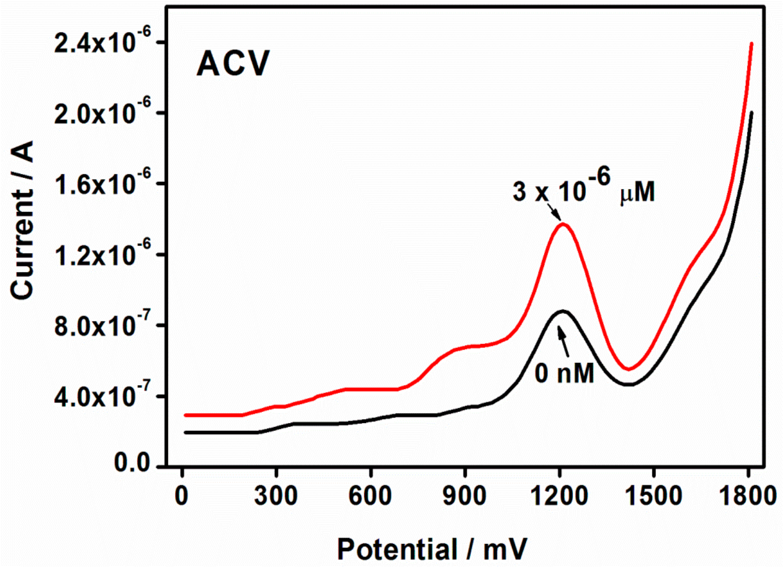

Figure S2. CV and ACV voltammograms of Au|G3PPT-co-P3HT sensor in the absence, and presence of PHE.

© 2015 by the authors; licensee MDPI, Basel, Switzerland. This article is an open access article distributed under the terms and conditions of the Creative Commons Attribution license (http://creativecommons.org/licenses/by/4.0/).
